# Results of an internal audit on the survival of patients with uterine sarcoma

**DOI:** 10.4274/jtgga.galenos.2018.2018.0083

**Published:** 2019-02-26

**Authors:** Florian Ebner, Saskia Wiedenmann, Inga Bekes, Wolfgang Janni, Nikolaus de Gregorio, Amelie de Gregorio

**Affiliations:** 1 Frauenklinik, HELIOS Amper Klinikum, Dachau, Germany; 2 Klinik für Frauenheilkunde und Geburtshilfe, Universität Ulm, Ulm, Germany

**Keywords:** Sarcoma, uterine, hysterectomy, fibroids, risk factors

## Abstract

**Objective:**

In the last 5 years there has been much discussion about the surgical procedure for uterine fibroids, and essentially, also uterine sarcoma. Still there exists no reliable presurgical diagnostic tool to differentiate between benign fibroids and uterine sarcomas. The aim of this study was to confirm the suspected association between intraoperative spread of tumor by morcellation and impaired outcomes in patients with sarcoma.

**Material and Methods:**

After the local ethics commission positively reviewed the study protocol, the oncologic database of our university hospital was retrospectively reviewed for patients with uterine sarcomas over a time period of 13 years (2002-2015). Data was extracted from the medical files and survival information was collected by contacting the patient’s general practitioners if last follow-up-status was older than 6 months. For the analysis, patients were split into two groups with either intrasurgical morcellation (M+) or no morcellation (M-) regarding information provided by the surgical report.

**Results:**

Data on 57 patients with uterine sarcoma were available for further analysis. The median age and body mass index of the patients was 63 years and 27 kg/m², respectively. The sarcoma subtypes were 25 leiomyosarcoma, 19 carcinosarcoma, 9 endometrioid stroma sarcoma, 3 adenosarcoma, and one case without further differentiation. In the majority, no morcellation was performed (M- group, n=44) and 51 patients received open surgery (3 laparoscopic, 1 vaginal, and 2 incomplete surgeries). The median time of follow-up was 31 months. The disease-free survival was 50.5 months and the Cox regression analysis showed a hazard ratio of 3.06 [no significant difference between the two subgroups (p=0.079; 95% confidence interval (CI): 0.9-10.6)]. The overall survival was found as 62.2 months and the Cox regression analysis showed a hazard ratio of 3.216 with a statistically significant difference between the two subgroups (p=0.013; 95% CI: 1.3-8.1).

**Conclusion:**

Despite the efforts to find a pre-surgical diagnostic tool, the clinical situation remains unsatisfactory. Overall sarcoma prevalence is low during the last 13 years at our university center, but morcellation occurred in a relevant portion of patients (13 of 57). If sarcoma is suspected or diagnosed then en-bloc resection of the uterus can prolong survival. Thus, morcellation of the uterus and not the surgical technique (en-bloc resection) is the prognostic factor and should be avoided in any suspicious case.

## Introduction

Uterine sarcomas are a rare malignant entity of the uterus (1,2)and are diagnosed in approximately 0.2-0.5% (2,3,4,5) of all cases of hysterectomies. The World Health Organization (WHO) classification differentiates between mesenchymal and mixed (mesenchymal and epithelial) tumors (6). Pure mesenchymal tumors are further differentiated into leiomyosarcomas (LMS), endometrial stromal sarcomas, and smooth muscle tumors of uncertain malignant potential, and mixed tumors are differentiated into adenosarcomas and carcinosarcomas (CS). CS along with mullerian mixed tumors, malignant mesodermal mixed tumors, and metaplastic carcinoma are considered a subclass of endometrial carcinoma (6). Generally, the prognosis of uterine sarcomas is unfavorable. Whilst the International Federation of Gynecology and Obstetrics (FIGO) stage Ia still has a 5-year survival rate of 84.3%, this dramatically decreases for stage II (43.6%), III (38.8%), and IV (19.8%) (7).

Clinical symptoms of this heterogenic tumor group might include uterine enlargement, bleeding, and pelvic pain, and are therefore rather unspecific and also common in many other gynecologic diseases (e.g. uterine leiomyomas). Blood parameters (serum lactate dehydrogenase, carcinoembryonic antigen, CA125, CA19-9, and CA15-3 (3,8), or presurgical imaging [ultrasound (US), magnetic resonance imaging (MRI), computed tomography (CT)] has room for improvement (3,9,10). Two case series for MRI scans found a positive predictive value of 52% (11) (negative predictive value 100%) and a specificity of 92% (12) to presurgically identify uterine sarcoma. Even positron emission tomography-CT is not capable of differentiating between benign uterine leiomyomas and malignant uterine sarcomas (13). US elastography case reports on the differential diagnosis of fibroids and sarcoma are being published (14), reporting a 'typical' mosaic pattern in sarcomas compared with a homogenous pattern in fibroids. 

Given the fact that myomas are a common finding in gynecologic patients, distinguishing between suspected malignant tumors and benign fibroids has great implications for clinical practice. Due to fertility aspects, hypermenorrhea, and urogynecologic symptoms, surgery in patients with fibroids is frequent. Surgical treatment of benign uterine leiomyoma is either focused on the removal of the myoma or the complete uterus. With increasing availability of laparoscopic equipment and surgical training, the number of open abdominal surgeries has decreased (15,16,17) over the last decades in favor of laparoscopic-assisted vaginal hysterectomy, total laparoscopic hysterectomy, or laparoscopic supracervical hysterectomy, which are offered to women who do not wish to bear children. A uterus-conserving approach is offered if family planning is not complete. The vaginal approach is limited by patient factors [e.g. body mass index (BMI), previous vaginal births/surgeries, size of uterus] and surgeons skills; however, the laparoscopic pathway is possible even with larger uterus size (18,19), increased BMI (20), offers rapid recovery, less blood loss (21) and a low complication rate (22). Laparoscopic surgery can be considered the standard surgical treatment of uterine leiomyomas, with large specimens often requiring morcellation to be removed through trocar insertion sites. This will increase the numbers of uterine sarcomas accidentally through morcellation. 

During morcellation, small visible and microscopic parts of the tissue may be dispersed within the abdomen. This might lead to peritoneal dissemination of tumor tissue (23). Based on the increased numbers of laparoscopic surgeries with subsequent morcellations, the rate of uterine sarcomas accidentally being morcellated will also increase. Given the general poor prognosis of uterine sarcomas (3,7,24) and the lack of sufficiently reliable preoperative diagnostic procedures to identify uterine sarcomas, this article tries to answer if accidental morcellation of uterine sarcomas in abdominal, vaginal or laparoscopic surgery has a negative impact on patients in terms of increased recurrence rates and/or decreased survival.

## Material and Methods

Our university cancer centre database has continuously collected data for all oncologic patients since 2002. This database was searched for patients with uterine sarcoma or CS including patients up to January 2016. Though the documentation input in the database is performed by well-trained and specialised personnel, the documenting of rare diseases might have been misclassified and not shown in the results. To maximise the results, the search was conducted by diagnosis or surgical procedure. The result list was then checked for agreement with the inclusion criteria. All patient files with a hysterectomy as surgical treatment at the certified gynaecologic oncology centre with age >18 years were included in this analysis. The available date were analysed retrospectively for tumour stage, histologic subtype, and route of surgery (open/laparoscopic or vaginal). The route of surgery was noted and patients were classified according to the surgical and pathology reports in uterine morcellation (M+) or en-bloc resection (M-). Morcellation in an intraabdominal bag was not performed. The disease-free survival (DFS) and overall survival (OS) were compared between these two groups. Living status and follow-up was provided by the routine annual cancer centre follow-up. If these data were not available, the patient’s general practitioner was contacted. Ethics approval (308/2012) was given by the Local Ethic Committee of Ulm University. 

Parameters for the statistical analysis using the SPSS software (IBM^®^ SPSS^®^ Statistics Version) were age at histologic confirmation of sarcoma (WHO classification), BMI, American Society of Anesthesiologists status, date and status of follow-up, primary tumour stage [tumour, node, metastasis (TNM), FIGO classification 2009], resection status (R0 or R1/2), receptor status (oestrogen, progesterone) and location of recurrence, as well as further treatments (e.g. radiotherapy, chemotherapy). Due to the small sample sizes, no analyses were performed based on the influence of morcellation regarding the different histologic subtypes.

Descriptive statistical analysis was used to determine average, median, standard deviation, minimum and maximum, likelihood, and percentiles. The OS/DFS were defined in months starting from the date of surgery to the last documented vital status/date of recurrence. Survival was analysed using Kaplan-Meier analysis, the log-rank test, and Cox regression. P<0.05 was considered statistically significant. Further multivariate testing for differences was performed using the Wilcoxon-Mann-Whitney test, univariate testing with the Fisher’s exact test, and the Mann-Whitney U test.

## Results

The database search identified 59 patients with sarcoma treated at Ulm University Hospital, Department of Gynecology and Obstetrics between 2002 and 2015. Two patients were excluded because no follow-up data were available. The average age of the remaining 57 patients was 63 years and their average BMI was 27 kg/m^2^. The histologic subtypes were LMS (n=25), CS (n=19), endometrial stroma sarcoma (n=9), high-grade sarcoma (n=3), and sarcoma without further classification (n=1). Twenty-nine patients were not TNM classified and only clinically staged, 15 patients were pT1, 10 pT2, and 5 pT3 after surgery. Detailed information on the two subgroups is presented in Table 1. Hormone receptors were negative or unknown in the majority of the specimens. Table 2 provides further patient and histologic details. It is noteworthy that our M+ subgroup had significantly larger tumours and patients with primary metastases.

The surgical access was abdominal in 51 patients, laparoscopic in 3 patients, and vaginal in one. Another two patients were considered incurable after the surgery had started. Three patients were started laparoscopically and converted to open abdominal surgery due to very large fibromas with adhesions (n=2) and once to repair a bladder lesion. Twenty-eight patients were considered R0, 5 patients had a microscopic tumour, and 24 patients could not be classified. Further details regarding the surgery are provided in Table 3. Further treatments included radiotherapy (n=11), chemotherapy (n=25), and no further therapy (n=10). Cause of death was known in 10 patients (sarcoma n=2, other causes n=8) with a further 15 patients deceased. The remaining 32 patients had a documented live status, who were used for further analysis. Disease recurrence was found in 20 patients. Recurrence occurred mostly as distant or a combination of distant and local metastases, followed by local and lymph node metastases. The uterus was removed without morcellation (M-) in 44 surgeries and 13 cases were considered morcellated (M+).

**Figure 1:**
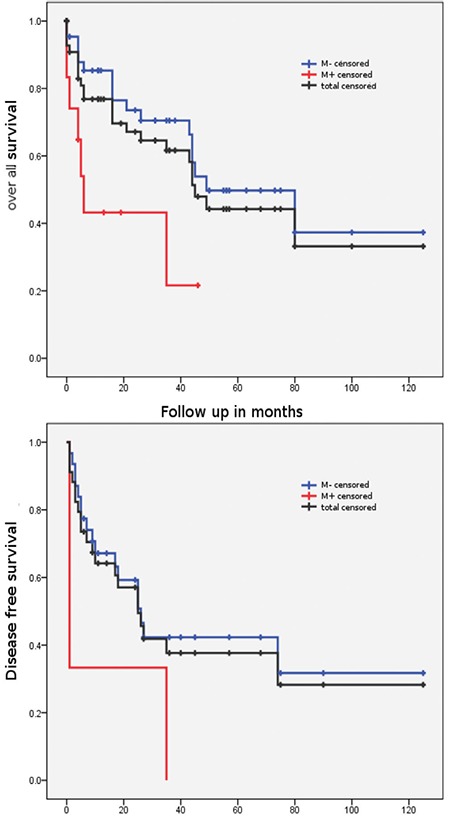
Disease-free survival and overall survival for all patients and the two subgroups. The disease-free survival difference M+/- is not statistically significant but should be considered clinically relevant. Patients with morcellation of the sarcoma (M+), no morcellation (M-)

The DFS of all patients was 50.5 months and Cox regressions analysis showed a hazard ratio of 3.06 without any significant difference between the two subgroups [12.3 months (M+) vs 54.9 months (M-); p=0.079; 95% confidence interval (CI): 0.9-10.6]. The OS was found as 62.2 months. Thereby, Cox regression analysis showed a hazard ratio of 3216 and was statistically significantly different between the two subgroups [19.2 months (M+) vs 69.2 months (M-); p=0.013; 95% CI: 1.3-8.1]. DFS and OS are presented in Figure 1.

## Discussion

Laparoscopic resection of uterine fibroids has been under scrutiny in recent years due to the lack of a preoperative diagnostic tool for uterine sarcoma. Reliable data on sarcoma incidence, diagnosis, prognosis, and further treatment are still rare. Prognosis for patients with uterine sarcoma is generally poor with a 5-year survival of 50% (25,26,27,28,29,30) (M+ vs M-: median OS 10.8 vs 39.6 months or 5-y OS 46% vs 73%) (26,31). Differences exist among subtypes and type of resection for survival. Endometrial stroma sarcoma and complete resection seem to be beneficial for the patient (32,33,34). Even in our small retrospective analysis, the results are in line with existing data on the recurrence pattern with mostly distant recurrence (35). 

Further data were published showing a decrease in survival if sarcomas were morcellated (31,36,37,38,39). The morcellation resulted in a tissue spill on various intraabdominal organs such as ovaries, liver, and omentum, and it did not matter which surgical technique (vaginal, laparoscopic or open) was used (40). Seidman et al. (41) published a reduced OS in patients with morcellation and LMS, but could not show this in other subtypes of uterine sarcoma. Similar results were published by other authors (26,42,43). Our data contribute to these conflicting results; DFS is not significantly different between the two surgical study groups – though there is a statistical trend indicating a disadvantage for the morcellated group. However the M+ subgroup had significant larger tumours and patients with primary metastases. However, our analysis shows a significant difference for OS, contrary to data published by Morice et al. (38). In their analysis, 123 patients were closely followed up and no significant difference in the 6-month recurrence rate was found between the two treatment groups (M- vs M+). However, the database includes various histologic subtypes (i.e. LMS, CS and endometrial stroma sarcoma with low and high-grade cases). The cases series by Liu et al. (44) indicates that there might be a very aggressive biologic subgroup, yet to be identified, due to the peritoneal metastasis in both surgical groups. 

Perri et al. (43) and George et al. (31) found a 3-fold increased risk for metastasis if the tumour was morcellated [hazard ratio (HR): 2.85; 95% CI: (1.05-7.5); HR: 2.95; 95% CI: (1.5-6.0)] (31,43), and a significantly shorter DFS (p=0.03 and p=0.002, respectively), which was similar to the results from Park et al. (45) who showed a significantly reduced OS and DFS in 56 patients with stage I and II LMS. Here, patients with a morcellation had more peritoneal and vaginal cuff metastasis. The most current published data indicate that patients with uterine LMS may have a shorter DFS and OS. Due to the low numbers in our analysis, the DFS difference of 42.6 months was not statistically different, but still should be considered clinically relevant. 

In early-stage low-grade endometrial stroma sarcoma, Park et al. (42) found a significantly shorter DFS but a longer, non-significant, 5-year OS for a morcellation subgroup. According to the authors the prolonged survival might be due to the more aggressive systemic therapy in case of morcellation and the short follow up. However, the incidence of accidental morcellation of uterine sarcoma seems to be low. In a large German monocentric retrospective study, the overall rate of uterine malignancies was 0.13% in more than 10,000 patients with morcellated uteri during laparoscopic-assisted supracervical hysterectomy. Thereby, the majority of malignancies were endometrial cancer (0.07%) with only 0.06% sarcomas [4 endometrial stromal sarcomas (0.04%) and 2 LMS (0.02%)] (46). As with any rare diseases, our retrospective database misses information on tumour classifications, follow-up, and most of all, the conclusions drawn from the analysis are restricted by the small number of cases. Unfortunately this also applies to most of the current literature regarding uterine sarcoma (47). 

Only a few authors clearly differ between the subtypes of sarcoma (31,42,43). Other studies, like ours, included various subtypes in the analysis. Some tumour variables cannot be provided by the pathologist. For example, the sarcoma size cannot be measured on a morcellated uterus. Thereby, this factor is a limiting point in study analysis and is important for appropriate assessment of tumour stage, and further required adjuvant therapy and can impact the ability to identify pathologic features for the determination of the tumour entity. In summary, a retrospective database will always miss certain information on the tumour that might be vital for further analysis. However, a prospective randomised trial with a known uterine sarcoma and deliberate morcellation on basis of the current data is unethical. Accordingly, the only possible and ethical way to increase knowledge on these rare diseases is through retrospective studies. 

Although this is a small, retrospective analysis, it includes all patients with uterine sarcoma over a time period of 13 years at a university hospital and investigates the impact of intraoperative morcellation. OS significantly differed between the intraoperative morcellation (M+) and whole-tumour resection (M-) subgroups. DFS also showed a clear, clinically relevant trend to impaired survival within the M+ group, but did not show a statistically significant difference. This is a common statistical issue with such small patient and follow-up numbers. Relapse mostly occurred as distant relapse. In contrast to some requests for abandoning morcellation in gynaecologic surgery, we recommend careful preoperative review and informed consent of intraoperative morcellation. This approach is in line with the Society of Gynecologic Oncology and the German Society for Gynecology and Obstetrics because purposeful use of morcellation allows less invasive surgery with reduced patients morbidity (48,49,50).

Although the overall numbers of patients treated with uterine sarcomas at our certified oncologic university centre is low, the rate of morcellated sarcomas (13 out of 57) underlies the clinical relevance of the topic. To address the clinical demand for improved identification strategies, we are currently performing a prospective liquid biopsy study on all patients with suspected LMS and storing the drawn blood samples for further investigation in our biobank. Possible target markers include vascular endothelial growth factor and cell-free RNA with evaluation of their use as prognostic and predictive factors. Other studies are also investigating possible mutations in sarcomas for personalized systemic treatment options (51).

Our data support resection of the entire uterus if any malignancy including sarcoma is suspected or known. For patients and physicians, a reliable presurgical test to eliminate the risk of uterine sarcoma is urgently needed.

## Figures and Tables

**Table 1:**
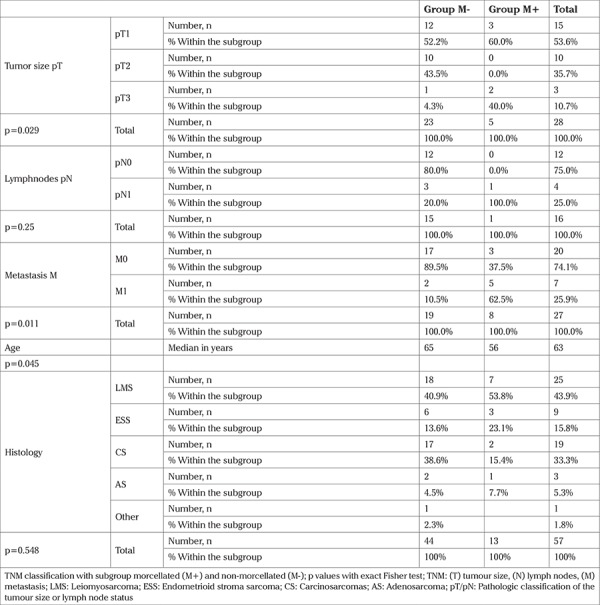
Patient and tumour details in the subgroups

**Table 2:**
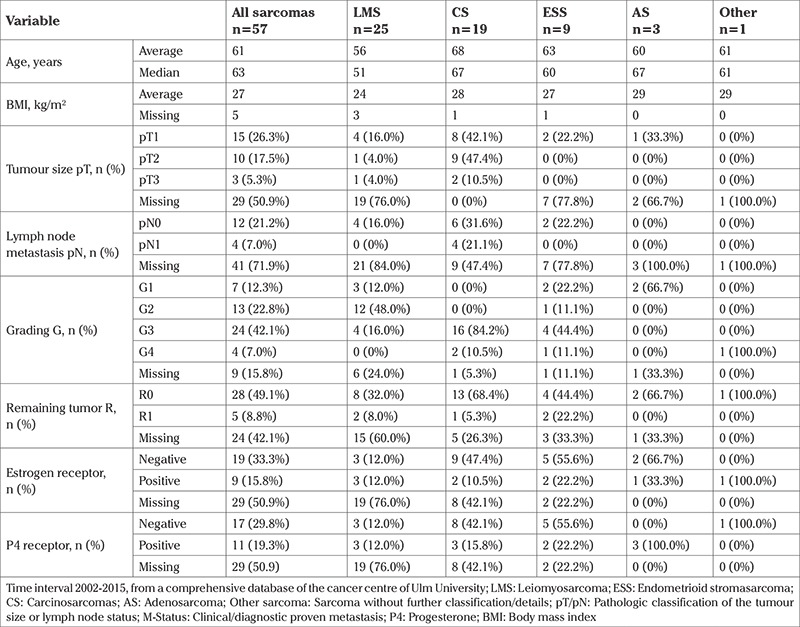
Patient and sarcoma details

**Table 3:**
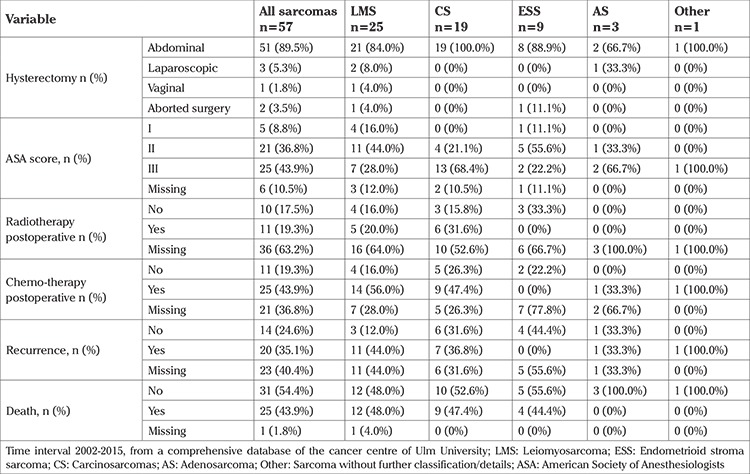
Surgical management, adjuvant therapy and outcome of patients with uterine sarcoma
